# Serial examination of cardiac function and perfusion in growing rats using SPECT/CT for small animals

**DOI:** 10.1038/s41598-019-57032-3

**Published:** 2020-01-13

**Authors:** Tomo Hiromasa, Junichi Taki, Hiroshi Wakabayashi, Anri Inaki, Koichi Okuda, Takayuki Shibutani, Kazuhiro Shiba, Seigo Kinuya

**Affiliations:** 1 Department of Nuclear Medicine, Kanazawa University Hospital, 13-1 Takara-machi, Kanazawa, Ishikawa, 920-8641 Japan; 2 Department of Physics, Kanazawa Medical University, 1-1 Daigaku, Uchinada, Kahoku, Ishikawa, 920-0293 Japan; 3 Department of Quantum Medical Technology, Institute of Medical, Pharmaceutical and Health Sciences, Kanazawa University, 5-11-80 Kodatsuno, Kanazawa, Ishikawa, 920-0942 Japan; 4 Division of Tracer Kinetics, Advanced Science Research Centre, Kanazawa University, 13-1 Takara-machi, Kanazawa, Ishikawa, 920-8641 Japan

**Keywords:** Biotechnology, Zoology

## Abstract

Few studies have evaluated myocardial perfusion and ventricular function in normal, growing rats. We, therefore, evaluated serial changes in cardiac perfusion and function during the growth of normal rats using single photon emission computed tomography (SPECT) with technetium (^99m^Tc)-sestamibi. Gated SPECT was serially performed in six normal rats. The left ventricular end-diastolic volume (EDV), end-systolic volume (ESV), stroke volume (SV) and ejection fraction (EF) were calculated with Quantitative Gated SPECT software. The perfusion distribution was calculated as the percentage uptake of each of the 17 segments using Quantitative Perfusion SPECT software. As expected, the body weight (BW) of the rats increased with growth, but their heart rates (HR) did not change over time. EF decreased very slowly over time and showed a negative correlation with BW. EDV, ESV and SV showed strong positive correlations with BW. There were no significant differences in the percentage segmental uptake in 13 of the 17 segments during growth, except for three basal and one apical segments. Therefore, a single normal database could be applied for the evaluation of perfusion abnormalities in rats of at least 8 to 28 weeks old.

## Introduction

Animal models of several heart diseases such as cardiac infarction, cardiomyopathy, myocarditis and myocardial injury due to anticancer drugs, hypertension, or diabetes mellitus have been widely used for assessing their etiology and developing treatment strategy for each disease^1–4^. As these diseases in human are clinically diagnosed with both non-invasive and invasive examinations including electrocardiogram, echocardiogram, magnetic resonance imaging (MRI), myocardiac perfusion scintigram, computed tomography (CT), cardiac angiogram and myocardial biopsy, these animal models have also been evaluated by the same methods in previous studies^5–7^. However, since animal hearts especially in rodents are very small and heart rate is very high compared with human, some non-invasive examinations used for human are hard to perform or have lower reproductivity and quantitativity for animal models. Moreover, since histopathological evaluation is usually difficult in animal models without euthanization, it precludes intra-animal follow-up for comparing histopathological change with therapeutic effect as well as long-term survivability.

Of many non-invasive diagnostic modalities for animals listed above, myocardial scintigram is reported to be well reproducible and highly quantitative^8,9^. This method is prevalent in clinical examination and therefore there are several established software for cardiac function analysis in human, such as, Quantitative Gated SPECT (QGS; Cedars-Sinai Medical Centre, USA), 4D-MSPECT (4DM; Invia Medical Imaging Solutions, USA) and Emory Cardiac Toolbox (Emory University, USA). They also have been used for analysing rodents’ cardiac function in previous studies. Gated SPECT permits simultaneous assessment of the distribution of myocardial perfusion and cardiac function [e.g., cardiac volume and ejection fraction (EF)].

Although relatively young rats were used in many previous reports, there is significant variability overall in the age of the rats used. This is a concern because cardiac perfusion may differ among age groups. For researchers planning cardiac experiments involving rats, specialised knowledge of the serial changes in cardiac function and perfusion related to natural growth will assist with selecting the appropriate age group of rats for particular experiments. In addition, there are few data regarding the long-time serial changes in cardiac function during the normal growth of rats^10,11^.

The aim of this study was to confirm the continuous change in cardiac function and perfusion distribution during growth using normal rats and gated SPECT with the tracer, technetium (^99m^Tc)-sestamibi and to compare them with the previous studies. Furthermore, we also evaluated whether histopathological changes such as fibrotic change would relate to functional change by aging.

## Results

### Phantom study

The ventricular phantom volume was calculated with QGS as 165 μL. The calculated ventricular volume was 89.2% of the real volume (184.9 μL). The mid-myocardium surface area was calculated as 199 mm2 (real surface area, 197 mm2), and the percentage uptake in each segment was calculated with QPS. The segmental % uptakes were: basal anterior, 75%; basal anteroseptal, 78%; basal inferoseptal, 73%; basal inferior, 63%; basal inferolateral, 69%; basal anterolateral, 71%; mid-anterior, 96%; mid-anteroseptal, 94%; mid-inferoseptal, 89%; mid-inferior, 88%; mid-inferolateral, 93%; mid-anterolateral, 94%; apical anterior, 96%; apical septal, 95%; apical inferior, 90%; apical lateral, 96%; and apex, 95%.

### Animal study

**Figure 1:**
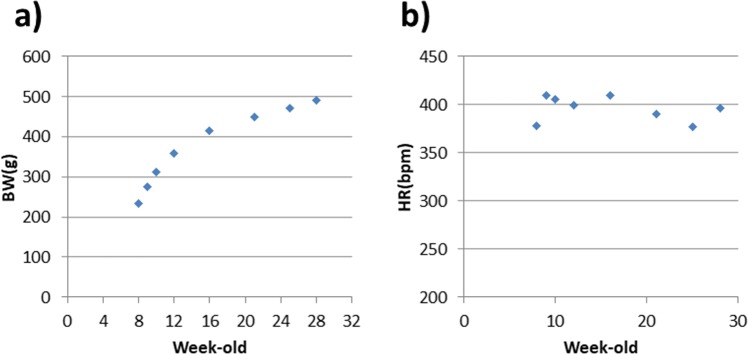
Changes in the body weight (**a**) and heart rate (**b**) during the normal growth of rats. Values represent the mean ± SD, n = 5–6.

The average BW of the six rats at 8 weeks old was 233 ± 2 g. The BW of the rats increased rapidly each week until they reached the age of 16 weeks (415 ± 15 g). After 16 weeks, the rate of increase in BW slowed. At the time of the final scan (28 weeks), the average BW was 492 ± 19 g (Fig. 1a, Table 1).

**Table 1:** BW, HR and various myocardial parameters were calculated with QGS software (mean ± SD, n = 5–6).

Age (weeks)	BW (g)	HR (bpm)	EDV (μL)	ESV (μL)	SV (μL)	EF (%)
8	233 ± 2	378 ± 39	316 ± 26	114 ± 8	202 ± 21	64.0 ± 2.2
9	276 ± 7	410 ± 18	349 ± 24	131 ± 11	218 ± 21	62.3 ± 3.1
10	312 ± 9	406 ± 24	393 ± 16	145 ± 20	248 ± 10	63.2 ± 3.8
12	358 ± 12	399 ± 23	434 ± 37	168 ± 17	266 ± 24	61.3 ± 2.1
16	415 ± 15	409 ± 16	479 ± 35	203 ± 14	276 ± 25	57.7 ± 1.8
21	450 ± 15	391 ± 20	530 ± 52	240 ± 21	290 ± 43	54.3 ± 4.1
25	471 ± 18	377 ± 14	497 ± 51	221 ± 35	277 ± 26	55.8 ± 3.7
28	492 ± 19	396 ± 22	503 ± 43	227 ± 11	276 ± 35	54.8 ± 2.7

BW, body weight; HR, heart rate; EDV, left ventricular end-diastolic volume; ESV, left ventricular end-systolic volume; SV, stroke volume; EF, ejection fraction.

The HR of the rats remained relatively constant, ranging from 370 to 410 bpm during growth with an average of 396 ± 25 bpm (Fig. 1b, Table 1).

### Functional measurements

The EDV of the rats increased between 8 and 16 weeks, but no significant differences were observed from 16 to 28 weeks. The ESV and SV increased at an almost equal rate, with no significant differences observed between 21 to 28 weeks for ESV and 10 to 28 weeks for SV. The EF decreased slowly over time.

**Figure 2:**
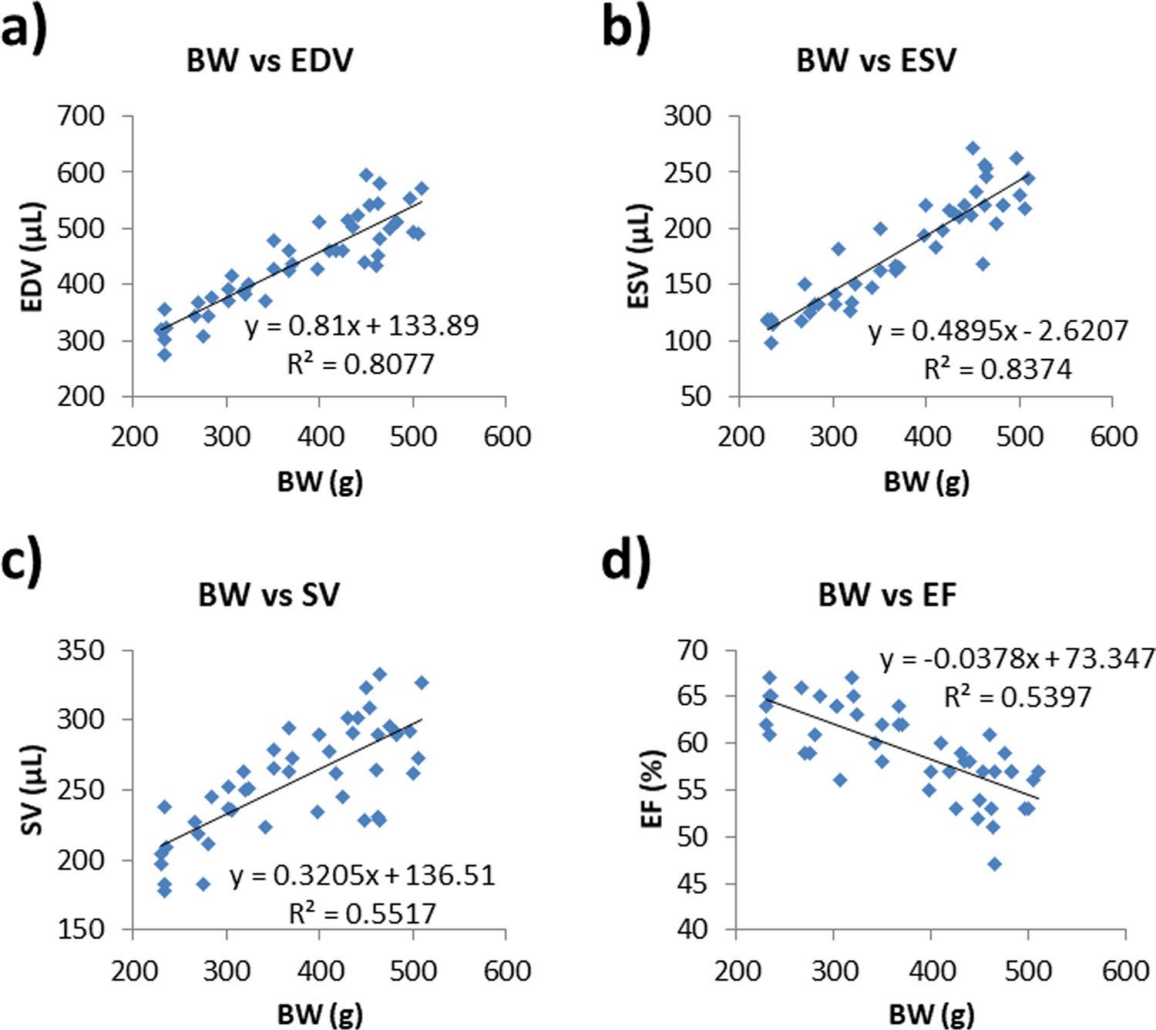
Correlations between BW, HR and each of the myocardial parameters were calculated with QGS software.

All the EDV (r^2^ = 0.8077, y = 0.81x + 133.89; p < 0.0001), ESV (r^2^ = 0.8374, y = 0.4895x + 2.6207; p < 0.0001), and SV (r^2^ = 0.55517, y = 0.3205x + 136.51; p < 0.0001) exhibited strong positive correlations with BW (y indicates EDV, ESV, or SV (μL), x indicates BW (g)) (Fig. 2). The EF showed a negative correlation with BW (r^2^ = −0.5397, y = −0.0378x + 73.347; p < 0.0001). There were no significant correlations between any of the functional parameters (EDV, ESV, SV or EF) and HR: HR vs. EDV (r^2^ = 0.0103; p = 0.5029), HR vs. ESV (r^2^ = 0.0027; p = 0.7308), HR vs. SV (r^2^ = 0.0; p = 0.3275) and HR vs. EF (r^2^ = 0.0066; p = 0.5916).

### Perfusion distribution

**Figure 3:**
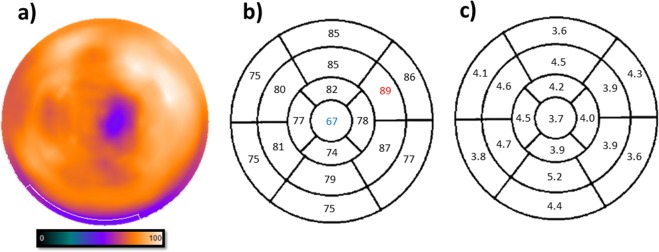
Representative perfusion maps of the rat heart during normal growth. (**a**) SPECT/CT image of perfusion in the rat heart using the tracer, technetium (^99m^Tc)-sestamibi. The average perfusion distribution (**b**) and % SD over the entire period (**c**) in each segment are indicated. The apical segment showed the lowest % uptake and the mid-anterolateral segment showed the highest % uptake.

The % uptake of each segment over the entire period is shown in Table 2. A polar map of the average % uptake distribution during growth is shown in Fig. 3. The apical segment showed the lowest % uptake (67 ± 3.7%), while the mid-anterolateral segment showed the highest % uptake (89 ± 3.9%). There were no significant differences in the % segmental uptake at any point in 13 of the 17 segments. Significant differences in % segmental uptake were, however, observed in the basal inferoseptal (10 vs. 21 weeks), basal inferolateral (12 vs. 28 weeks), basal anterolateral (8 vs. 28 weeks, 10 vs. 28 weeks) and apical septal (8 vs. 21 weeks) segments. A proportion of these changes in the % uptake in some of the basal segments tended to occur because of fluctuations in basal myocardial edge detection. In the non-basal segments, the % uptakes were quite stable.

**Table 2:** Average % uptake in each segment in normal, growing rats from 8 to 28 weeks old.

	All	8 w	9 w	10 w	12 w	16 w	21 w	25 w	28 w	Phantom
Basal anterior	85	86	86	85	86	86	81	83	84	75
Basal anteroseptal	75	78	76	76	75	76	72	78	74	78
Basal inferoseptal	75	78	74	^*^79	74	75	^*^72	77	73	73
Basal inferior	75	75	75	76	73	75	73	79	78	63
Basal inferolateral	77	79	76	78	^**^74	76	75	77	^**^80	69
Basal anterolateral	86	^¶^90	88	^#^89	86	88	85	84	^¶#^82	71
Mid-anterior	85	84	85	88	85	85	86	84	88	96
Mid-anteroseptal	80	80	79	81	79	81	77	84	79	94
Mid-inferoseptal	81	83	79	84	79	83	80	84	79	89
Mid-inferior	79	78	76	79	77	80	79	82	78	88
Mid-inferolateral	87	89	86	90	83	88	86	88	90	93
Mid-anterolateral	89	89	89	90	88	89	90	90	85	94
Apical anterior	82	82	80	84	81	82	84	82	82	96
Apical septal	77	^‡^81	76	80	76	79	^‡^73	76	76	95
Apical inferior	74	73	72	76	71	77	74	76	72	90
Apical lateral	78	78	77	81	76	78	78	82	78	96
Apex	67	67	65	69	65	68	66	66	68	95

^*,**,¶,#,‡^Statistically significant pairwise comparisons with P values < 0.05.

### Histology

**Figure 4:**
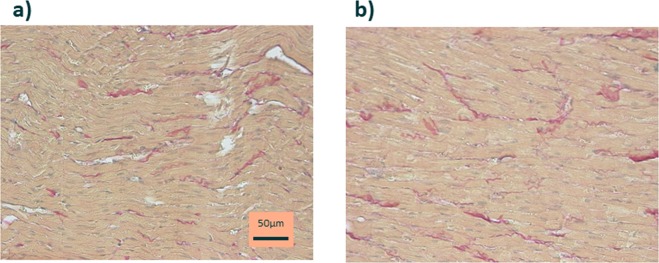
Elastica van Sirius red-stained myocardium of an 8 weeks old Wistar rat (**a**) and a 25 weeks old Wistar rat (**b**). In their myocardium, minimal areas of collagen deposition (red) are dispersed throughout myocardial tissue in both groups of rats and not different. (**a**,**b**) original magnification ·400.

Eight and 25 weeks old rat’s heart sections were stained with Miller’s Elastica van Sirius red stain (Fig. 4). The average fibrosis (%area) was 2.83 ± 0.45% at 8weeks (n = 6) and 3.00 ± 0.24% at 25weeks (n = 6). There was no significant difference in the amount of fibrosis at 8weeks versus 25weeks (p = 0.78).

## Discussion

In this study, we observed the cardiac functional changes that occurred during the growth of relatively young rats from 8 to 28 weeks old. The data showed that, whereas the size of the rat hearts increased greatly, the EF remained relatively stable but decreasing gradually, and the perfusion distribution was quite stable over time.

Our study showed that the EF was 60–65% from 8 to 10 weeks old and then declined gradually to around 55% at 21 weeks old and remained stable thereafter. In a previous study, EF was 60% and less at 8 weeks old and 40% and less at 50 weeks old, which is consistent with our results indicating that EF declines with growth^10^. On the other hand, in relatively old rats, no significant changes were observed in EF at 6 months old and 36 months old^11^. We suggest that the cause of the slow decline in EF in our study is not due to myocardial fibrosis because there was no significant difference in the degree of fibrosis between 8 weeks and 25 weeks old rats. Bisi *et al*. reported that insulin-like growth factor as well as growth hormone is able to promote inotropic effects in both humans and other animals^12^. In human study, Cain *et al*. reported that left ventricular EF demonstrated a decline with age; EF in the adolescent group was higher compared to adult subjects (0.72 ± 0.05 vs. 0.61 ± 0.07, p < 0.001). They speculated that the higher EF in younger humans may possibly, in part, reflect their higher growth hormone levels^13^. So, we speculate that the cause of the slow decline in EF in our study is the effect of not pathological organic change but intrinsic physiological change such as hormones.

We could not perform our study in rats older than 6 months because these rats were too large to enter into the gantry of our scanner. In contrast with the results for cardiac function, the data on the distribution of the regional perfusion showed almost no change from 8 weeks old to 28 weeks old, which indicates that any age-related changes of the myocardial perfusion distribution have not yet occurred in up to 28-week-old rats at least. Therefore, evaluation of relative blood flow can be possible using a standard normal perfusion map with ^99m^Tc-sestamibi in 8- to 28-week-old rats. Actually, Fig. 3 could be used as a single normal database. Among all 799 segmental % uptake data obtained in this study, only 18 segments’ data (2.25%) were out of the mean ±2 SD.

The differences in the % uptake of perfusion between the phantom and rat heart were not small, especially for the mid to apical segments. There are several factors that might have caused the difference. The major factors might have been related to the presence or absence of the absorber around the heart and the extracardiac background activity. Differences in the shape of the heart might have also influenced the calculated distribution of the tracer. Pulsation also affects the calculated tracer uptake, particularly in the apex. Overall, the distribution of tracer uptake in the rat showed a similar pattern to that observed in humans, i.e., low apical uptake with the mid-anterolateral uptake being the highest^14^.

In our study, the apical segment showed the lowest perfusion during growth. On the other hand, Strydhorst *et al*. reported that the lowest perfusion was shown in apical lateral area, using the other commercial software, 4DM, for the analysis, in 12 male Sprague-Dawley rats using nanoSPECT small animal camera (Bioscan, USA)^8^. We consider these distribution differences have been due to not only attenuation artefact and partial volume effect due to particular SPECT system, but also a cardiac or respiratory motion blurring effect and methodological differences including rat’s body position at data acquisition. Especially one concern is the differences in the measured parameters when analysed by different software packages. One software package should be chosen and consistently used to analyse a set of data. The characteristics of the particular analysis software should be evaluated before using the software for either an experiment or a phantom study.

Over all, the results of this study provide the initial data for a normal reference database for the evaluation of pathological cardiac models. This is necessary for weight- and age-appropriate comparisons of cardiac function parameters between normal and myocardial injury models or pathological models. In injury models, body weight (BW) gain is often slower than in the normal rat. In such cases, it may be better to compare the disease model with normal rats based on weight rather than age, because BW and cardiac functional parameters showed significant correlations.

### Limitations

In this study, we chose inhalation anesthesia with isoflurane for animal imaging, considering the stability of anesthesia and the effect on cardiac function. It has been reported that anesthetic had some influence on cardiac function and myocardial perfusion^15^. For intraperitoneal anesthesia, ketamine/xylazine have been reported that those significantly affect cardiac function such as heart rate, EF and EDV, and pentobarbital has also been reported to decrease EF compared with isoflurane. On the other hands, it has been reported that isoflurane increases myocardial blood flow depend on the concentration without changing HR^16,17^, which is considered to be acceptable for analyzing kinetic analysis of cardiac function such as Gate SPECT. However, the possibility of changing the perfusion distribution by isoflurane was not previously mentioned and was not examined in our present study. We should consider that whether the perfusion distribution changes depending on the type and concentration of anesthetic.

Our data were obtained using only six rats, but the data are likely to be reliable because the standard deviations (SD) in the data were generally small and all measurements showed high reproducibility. This is likely attributable to the high spatial and temporal resolution of SPECT/CT^18,19^. With a multi-pinhole collimator, ECG synchronisation could be performed successfully even at a heart rate of 400 bpm.

We started initial data acquisition at 8 weeks old, since 8 to 12 weeks old rats are commonly used to create disease models. After model creation, follow up period depends on the particular disease model. So we continued data acquisition as long as possible until the rat size limit the experiment.

Myocardial perfusion semi quantification is the unique feature of SPECT. However, heart functions assessment may require comparison with other modalities.

## Conclusion

We analysed rat cardiac functional parameters and the distribution of relative cardiac perfusion in normal, 8- to 28-week-old rats using the ^99m^Tc-sestamibi tracer and gated SPECT/CT with the QGS and QPS software for analysis. The EDV, ESV and SV increased continuously over time and showed a significant positive correlation with BW. The EF showed a gradual reduction during growth and a significant negative correlation with BW. For the perfusion distribution, the apical segment showed the lowest % uptake and the mid-anterolateral segment showed the highest % uptake over the test period. In 13 of the 17 segments, there were no significant differences observed in % segmental uptake over time. Thus, other than in the basal area, the distribution of perfusion in normal rats was quite stable during growth. Therefore, a single normal database that shown in Fig. 3 could be applied to the evaluation of perfusion abnormalities at least for 8- to 28-week-old rats.

## Methods

### Phantom study

A phantom study was performed using the Quantitative Gated SPECT (QGS; Cedars-Sinai Medical Centre, USA) and Quantitative Perfusion SPECT (QPS; Cedars-Sinai Medical Centre) software packages to measure the phantom volume and radionuclide distribution. We used an original rat heart phantom of the left ventricle with a ventricular volume of 184.9 μL, 10 mm in length along the long axis, 5 mm in diameter, with a myocardium compartment thickness of 1 mm and a mid-myocardium surface area of 197 mm2 (HokurikuEP, Japan), filled with 7 MBq of ^99m^Tc (the myocardium volume of this phantom was 0.22 mL, and the radionuclide concentration was 32.1 MBq/mL). The apical portion was hemispherical. Data were acquired with a small-animal SPECT system (VECTor/CT, small-animal camera; MILabs, The Netherlands) equipped with the General Purpose Rat and Mouse (GPRM) collimator (MILabs). The sensitivity of this collimator for ^99m^Tc was 1851 cps/MBq, and the full width at half maximum was 1.38 mm. Total imaging time was 20 min in the list mode.

### Animal studies

All experimental procedures involving animals were conducted in accordance with the institutional guidelines set by the Institute for Experimental Animals, Kanazawa University Advanced Science Research Center and the experimental protocol was approved by the Committee for Animal Experiments of Kanazawa University on May 16, 2018 (approval number: AP-173846). Six male, 8-week-old Wister rats were investigated in this study. The rats were imaged at 8, 9, 10, 12, 16, 21, 25 (n = 6) and 28 (n = 5, one of them died before scanning because of an anaesthesia error) weeks old using the VECTor/CT system fitted with the GPRM collimator. All rats received 185 MBq of ^99m^Tc-sestamibi via the tail vein 20 min before ECG gated SPECT/CT, to reduce physiological uptake in the liver. Animals were scanned for 15 min in the prone position under anaesthesia with 2% isoflurane inhalation. Cardiac cycles were divided into 16 frames.

### Image data analysis

Phantom and rat heart data were acquired in the list mode and the photopeak windows were set after acquisition (140 keV, 20% width). We employed triple energy window scatter correction in both the phantom and animal experiments. Data were reconstructed using pixel-based ordered subset expectation maximisation, without attenuation correction, using 16 subsets and 6 iterations for the phantom and rats^20^. Data were filtered with the 1-mm size Gaussian filter as the post filter. The original voxel size (0.8 × 0.8 × 0.8 mm) was multiplied by 10 in each side to approach that of the human reference heart for analysis with the QGS and QPS software^21,22^.

**Figure 5:**
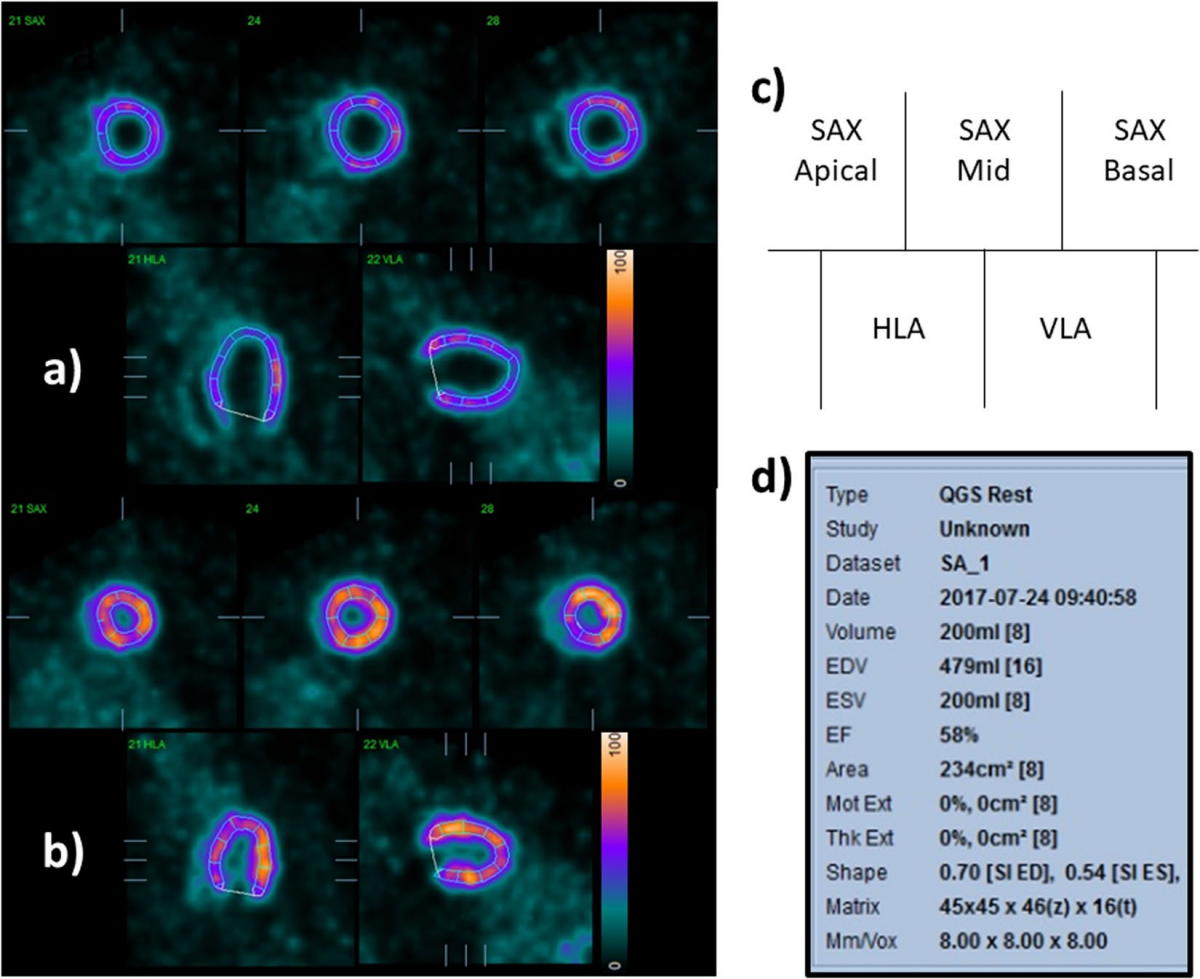
Representative slices of the rat hearts were analysed with QGS software. (**a**) Endo-diastolic phase, (**b**) endo-systolic phase, (**c**) image orientations in (**a**,**b**,**d**) parameters calculated with the QGS software. SAX, short-axis view; HLA, horizontal long-axis view; VLA, vertical long-axis view.

Using QGS software, we calculated the LVEF, EDV, ESV and SV. The QGS software algorithm operated in three-dimensional space and used gated short-axis image volumes. It segmented the left ventricle, estimated and displayed the endocardial and epicardial surfaces for all gating intervals in the cardiac cycle, calculated the relative left ventricular cavity volumes and derived the global EF from the EDV and ESV, all without operator interaction (Fig. 5)^23,24^.

The QPS software was used with a 17-segment system as previously described in a clinical study that examined the serial change in the % uptake of the tracer in each segment^25^. The relative segmental average count level was defined as the count ratio value given by the QPS software using the standard normalisation factor method with the 80^th^ percentile of the maximum count in pixels.

### Histology

We selected 8 weeks (n = 6) and 25 weeks (n = 6) old rat’s heart tissues. Tissues were fixed in 4% buffered formalin and processed for paraffin embedding. Left ventricular short axial sections (5 μm thickness) were cut from middle area. Selected sections were stained with Miller’s Elastica van Sirius red stain according to standard procedures, for analysis of fibrosis. Using a microscope with x400 images, the percent area of fibrosis was calculated by using imageJ software (Version 2.0.0; National Institutes of Health, USA) in each image (5 fields/rat).

### Statistical analysis

Results are expressed as the mean ± SD. A threshold *P* value of <0.05 was considered significant. Statistical analyses were performed with JMP Software (Version 11.2.0; SAS Institute, USA). The relationships among EDV, ESV, SV, EF, BW and HR were analysed by linear regression (r^2^). Statistical differences in the % uptake of myocardial perfusion each week were calculated with the T test using the Turkey–Kramer method. Statistical analysis in the average % area of fibrosis at 8weeks and 25 weeks was performed using the unpaired T test.

### Compliance with ethical standards

All applicable international, national and/or institutional guidelines for the care and use of animals were followed.
